# Extension of the mutation spectrum of *PAX6* from three Chinese congenital aniridia families and identification of male gonadal mosaicism

**DOI:** 10.1002/mgg3.481

**Published:** 2018-10-17

**Authors:** Zhouxian Bai, Xiangdong Kong

**Affiliations:** ^1^ Genetic and Prenatal Diagnosis Center The First Affiliated Hospital of Zhengzhou University Zhengzhou China

**Keywords:** congenital aniridia, double mutation, frameshift mutation, male gonadal mosaicism, *PAX6*

## Abstract

**Background:**

Congenital aniridia is a severe autosomal dominant binocular developmental disorder, the primary feature of which is congenital absence or hypoplasia of the iris. *PAX6* is the main disease‐causing gene of congenital aniridia; inheritance is autosomal dominant. But the current mutations do not fully explain this disorder.

**Methods:**

We investigated the mutation profile of genes related in three Chinese families with congenital aniridia through targeted sequencing technology. And we validated the candidate variants by PCR‐based Sanger sequencing. Different degree impairments of islet function were observed in the patients with aniridia by carbohydrate tolerance butter and insulin release tests in our study.

**Results:**

We identified four novel mutations of *PAX6* from three Chinese families with congenital aniridia, which included heterozygous double mutation c.879_880delCA (p.S294Cfs*46) and c.1124C>G (p.P375R) in Family 1 with three patients, heterozygous frameshift mutation c.308delG (p.P103Qfs*21) in Family 2 with one patient, and c.1192delT (p.S398Pfs*126) in Family 3 with two patients. The three frameshift mutations of *PAX6* are co‐segregated with the aniridia from controls in the families, but the novel missense mutation is not co‐segregated with the phenotype. The frameshift mutations in Family 1 and Family 2 have effects to truncate the protein, but the frameshift mutation in Family 3 will prolong it. We confirmed the phenomenon of male gonadal mosaicism of *PAX6* by the sequencing of two linked novel mutations in Family 1. Most of the patients with isolated aniridia have different degrees of islet damage through related clinical tests.

**Conclusion:**

It is therefore noteworthy that we found different types of pathogenic mutation, which have effects of truncating or prolonging protein leaded by frameshift mutation. Our results of this study extended the pathogenic mutation spectrum of *PAX6* for congenital aniridia and demonstrated the male germline chimerism by molecular experiments.

## 1 INTRODUCTION

Congenital aniridia is a severe autosomal dominant binocular developmental disorder, and the primary feature is congenital absence or hypoplasia of the iris (Lim, Kim, & Kim, 2017). Aniridia usually occurs in both eyes during infancy, which can lead to reduced visual acuity and nystagmus. The symptoms also include cataract, glaucoma, and corneal abnormalities. Congenital aniridia is mainly associated with pathogenic variants in the *PAX6* [OMIM:607108] gene, a master control gene for eye development, located on chr11p13 (Ton et al., 1991). The *PAX6* is a highly conserved transcription factor that played an important role in the development of human eyes and islet cells (Glaser et al., 1994). Related animal studies indicate that *PAX6* gene is critical for islet cells, which involved in insulin synthesis and secretion through regulating the proteins encoded by key genes during transcription progress (Gosmain et al., 2012). Whether the isolated congenital aniridia patients could be found combined with abnormal glucose tolerance or early onset diabetes needs more observations.

To in‐depth comprehend the mutation profile of genes related and clinical features in congenital aniridia, we collected three Chinese families with six patients in total and screened them using targeted sequencing technology. And we also investigated the clinical phenotypes of these patients so as to understand the mutation effects. To supplement and perfect, the genetic profile of congenital aniridia may improve our understanding of heterogeneity and severity of the disease.

## 2 MATERIALS AND METHODS

### 2.1 Ethical compliance

Clinical investigations were conducted according to the Declaration of Helsinki, and the study was approved by the institutional review board of the Medical Ethics Committee of the First Affiliated Hospital of Zhengzhou University.

### 2.2 Subjects

Three Chinese congenital aniridia families were recruited for this study. Samples were obtained with written informed consent. The aniridia patients came for medical and genetic consultations in our hospital in 2017, and 4 ml peripheral blood was drawn from the persons in the three congenital aniridia families, respectively. We collected 2 ml semen of the proband's father in the Family 1 on the principle of voluntariness. Amniotic fluid of the mother in the Family 3 was extracted for prenatal diagnosis. The clinical material of aniridia patients in these three different families was collected when they got medical service at the clinic.

### 2.3 Clinical presentations

#### 2.3.1 Family 1

**Figure 1:**
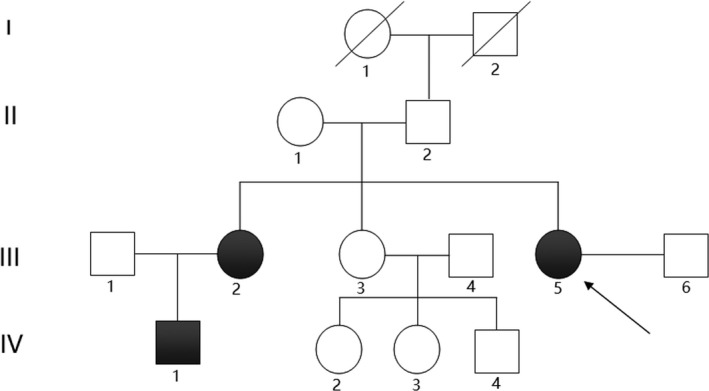
Pedigree of the congenital aniridia Family 1

**Figure 2:**
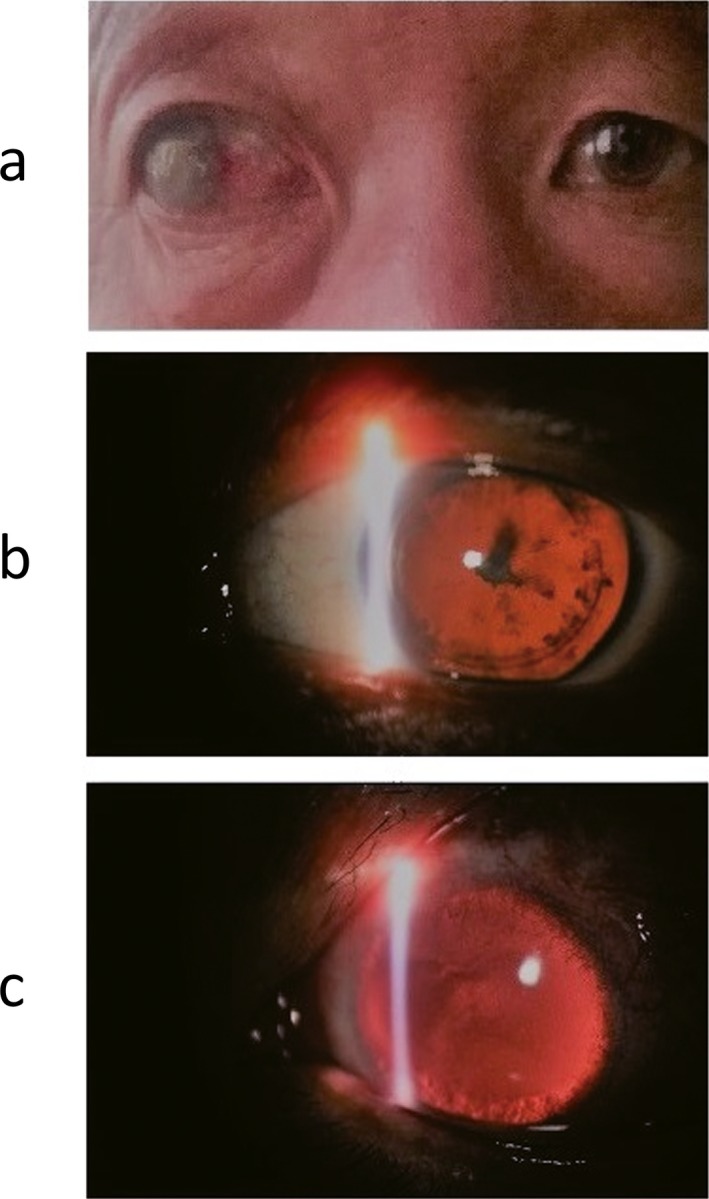
Eye pictures of partial members of the Family 1 (provided by the proband). Part a is the binocular photograph of the proband's father (II2, Figure 1) as control, whose right eye is traumatic blindness and left eye is normal; Part b is the slit‐lamp biomicroscopy photography of the left eye of the patient (III5, Figure 1) as proband, who combined with cataract before cataract surgery; Part c is the slit‐lamp biomicroscopy photography of the left eye of the patient (IV1, Figure 1)

The pedigree of the Family 1 is shown in Figure 1, and they come from Xinxiang, Henan Province. The general situation of the seven persons in this family is shown in Table 1. Three members in the Family 1 are affected with aniridia. Patients III2, III5, and IV1 all underwent aniridia, and the proband (III5) has also undergone glaucoma after cataract surgery. The proband (III5) was found suffering from congenital aniridia with cataract at the age of 17 years old. The patient III5 was diagnosed with diabetes recently, who has worst phenotypes of aniridia and nystagmus that both of the eyes involved. The slit‐lamp biomicroscopy photography of the left eye of patient III5 is shown in Figure 2. Patient III2 has cataract combined with aniridia and both eyes involved, and patient IV1 has no other phenotypes with his eyes, and this may be just because he was young. The patients III2 and IV1 were found with impaired glucose tolerance by glucose tolerance test due to the suggestion of the proband's diabetes. The other nine members of the family are with normal phenotypes. The proband's father (II2) has traumatic blindness of right eye and normal left eye. The three patients with aniridia have different clinical feathers. The proband provided us three photographs of the eye of partial members of Family 1 (Figure 2). The picture intuitively shows the situation of eyes of members II2, III5, and IV1 in Family 1. Patients with aniridia in this family showed heterogeneity of clinical manifestations.

**Table 1:** Overview of clinical phenotypes in the Family 1

Clinical manifestation	II1	II2	III1	III2	III3	III5 Proband	IV1
Gender	F	M	M	F	F	F	M
Age	56y	56y	33y	32y	27y	26y	13y
Left vision	Normal	Normal	Normal	Worse	Normal	Worse	Worse
Right vision	Normal	Traumatic blindness	Normal	Worse	Normal	Worse	Worse
Aniridia	NO	NO	NO	YES	NO	YES	YES
Both eyes involved	NO	NO	NO	YES	NO	YES	YES
Nystagmus	NO	NO	NO	YES	NO	YES	YES
Cataract	NO	NO	NO	YES	NO	YES	NO
Glaucoma	NO	NO	NO	NO	NO	YES	NO
Diabetes	NO	NO	NO	Impaired glucose tolerance	NO	YES	Impaired glucose tolerance

#### 2.3.2 Family 2

**Figure 3:**
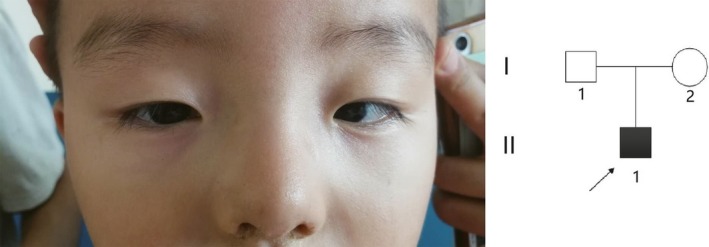
Eye picture of the proband (II1) and pedigree of the congenital aniridia Family 2

**Figure 4:**
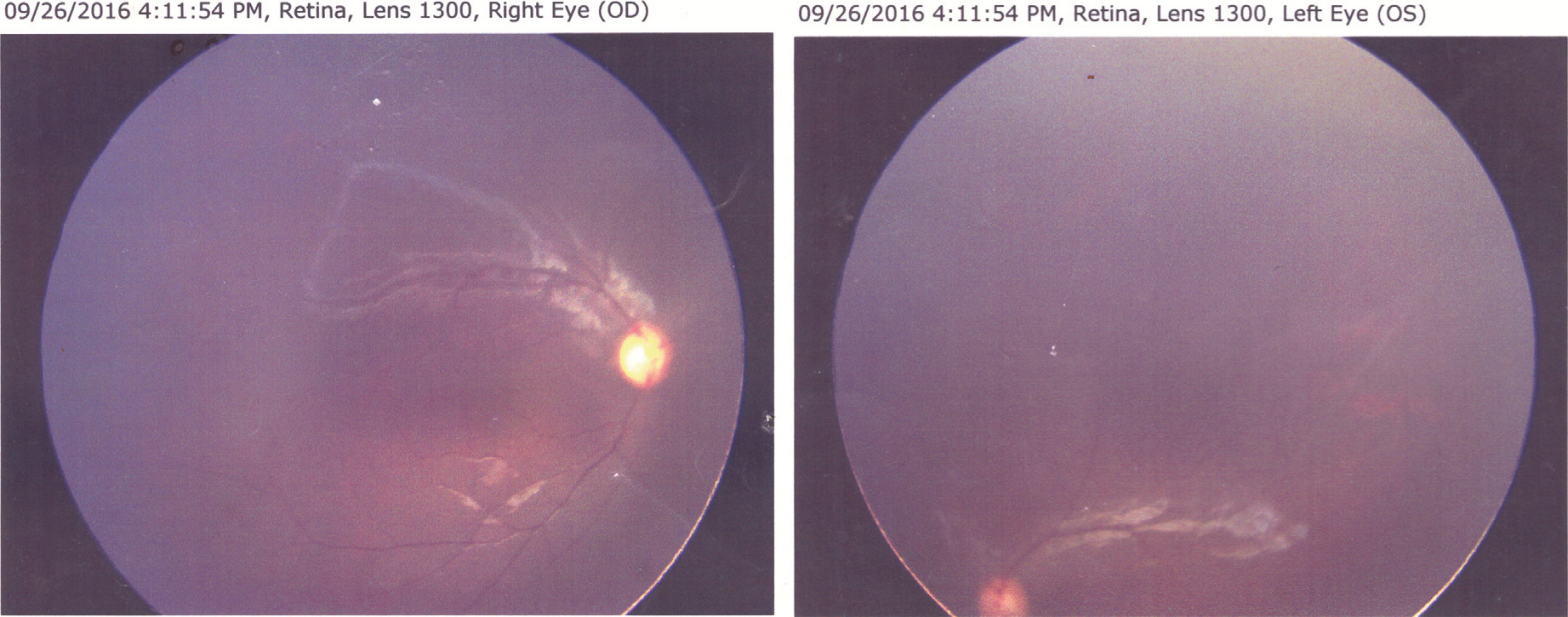
Fundus image of the proband (II1) with aniridia in the Family 2

The proband's eyes and pedigree of the congenital aniridia in Family 2 are shown in Figure 3. They come from Zhongmu, Henan Province. The patient with aniridia, 3 years old, has no cataract and glaucoma, but has mild ptosis of upper eyelid. The parents of the proband have normal phenotypes. The child was born after the normal period of gestation by cesarean section. The child's eyes were open 3 days after birth, and his parents found him afraid of light. Ophthalmologic examination revealed that the child has the absence of iris but with transparent cornea and crystal. The ocular fundus examination showed that (Figure 4) both optic disks of the eyes were clear and red, and the blood vessels were normal. There was no obvious choroid defect and no obvious abnormalities in the fundus. Macular region structure was existence. The patient developed horizontal nystagmus at 2 years old, and the result of cycloplegic refraction showed the child with farsightedness (OD: +3.00DS; OS: +3.00DS).

#### 2.3.3 Family 3

**Figure 5:**
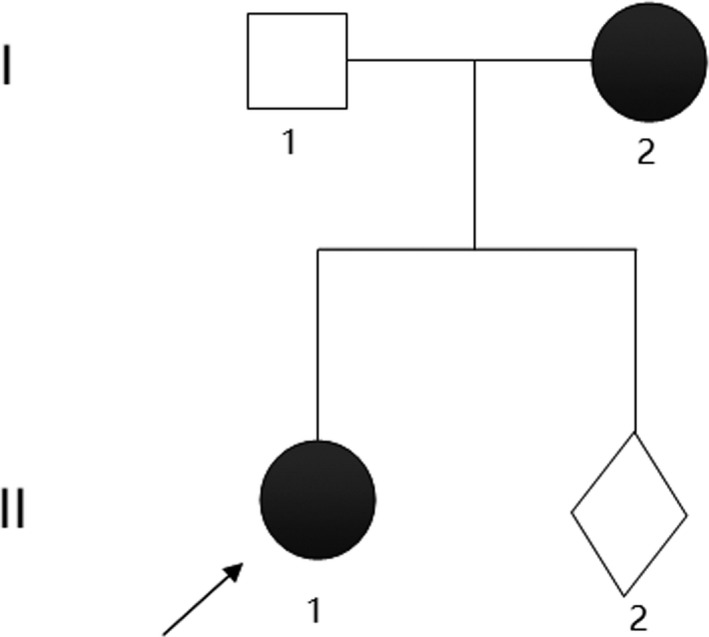
Pedigree of the congenital aniridia Family 3

The pedigree of the Family 3 is shown in Figure 5, and they come from Shijiazhuang, Hebei Province. The proband is 14 years old and has congenital aniridia combined with cataract. Her father has the normal phenotypes, and her mother has the same phenotypes like her. Amniotic fluid of the mother was collected to extract DNA for prenatal diagnosis.

### 2.4 Genetic test

Genomic DNA was extracted from EDTA‐treated blood samples using Blood DNA Midi Kit D3494 (Omega Bio‐tek, USA) through nucleic acid automatic extraction equipment (Eppendorf epMotion 5075 m, Germany). Genomic DNA of semen was extracted using E‐Z 96^®^ Tissue DNA Kit D1196 (Omega Bio‐tek, USA). Amniotic fluid cell DNA was extracted and cleaned using QIAamp Blood DNA Midi Kit (250, Germany) and Genomic DNA Clean & Concentrator (Zymo Research, USA).

A customized panel (MyGenostics, Beijing China) was designed to capture 662 known genes (Supporting information Table S1) related with ophthalmology phenotypes in order to detect the genetic cause of the congenital aniridia families. The panel sequencing was conducted on the Illumina NextSeq500 system in our clinical laboratory. Version GRCh37 is the human reference genome used for short reads mapping. PCR‐based Sanger sequencing was used to validate the variants which are chosen as disease‐causing mutations through NGS. The carrying situation of the novel mutations of their family members was also tested by Sanger sequencing. The PCR primers (Table 2) were designed by GeneTool software. Capillary electrophoresis apparatus (ABI 3130xl, USA) and dGTP BigDye^®^ Terminator sequencing kit (ABI, USA) were used for Sanger sequencing.

**Table 2:** Primer pairs of the novel mutations of *PAX6*

Gene exon	Transcript	The sequence‐specific primers of upstream (5′‐>3′)	The sequence‐specific primers of downstream (5′‐>3′)	Product length(bp)
*PAX6*‐exon10	NM_000280	GGCTCGACGTAGACACAGTGCT	TGAGGGCAAGAGAAATGACAGTA	293
*PAX6*‐exon12	NM_000280	GGGCTGTGGCTGTGTGATGTGT	ACACGCCCTCCCATAAGACCAG	303
*PAX6*‐exon6	NM_000280	CCAACGGATGTGTGAGTAAAAT	GCAGGGAGAGGACACAGACTAA	323
*PAX6*‐exon13	NM_000280	TTTGTATTCCATGTCTGTTTCTC	AGCCATTTTTCTTTCTTTCCTG	253

Goldeneye^™^ 20A kit (peoplespot co.Ltd, Beijing, China) was used for paternity test of the members (II1, II2, III2, and III5) in the Family 1. PCR products were measured by the automatic electrophoresis instrument (QIAxcel system, Germany) in our laboratory.

### 2.5 Population control

We collected the exome sequencing data of 22 patients and panel sequencing data of 44 patients without eye disease who made medical consultations at our department. We also applied for the whole genome sequencing data of public database ADNI (Alzheimer's Disease Neuroimaging Initiative, http://adni.loni.usc.edu) and downloaded the sequencing data of 809 subjects without oculopathy authorized by ADNI. We extracted the genotypes of corresponding positions of the novel mutations from the sequencing data as population control. The sequencing data of 875 subjects that used as population control were approved by the medical ethics committee of the first affiliated hospital of Zhengzhou University.

### 2.6 Functional prediction analysis

Retrieving the candidate pathogenic mutation sites in the public database included dbSNP, 1,000G, and ExAC, and checking them whether been recorded. We also plan to retrieve the candidate sites in the Human Gene Mutation Database at the Institute of Medical Genetics in Cardiff (HGMD) Professional to ensure their pathogenicity. PhyloP and PhastCons software were used to analyze the conservation of corresponding amino acid sequence of the missense mutated locus. Pathogenic analysis was conducted by SIFT, PolyPhen_2, and Mutation t@sting online tools. We also analyze the second structure, disorder region, and mutation effect of the missense mutated site by PredictProtein online tool (Schlessinger, Yachdav, & Rost, 2006).

### 2.7 Islet function‐related clinical examinations

We investigated islet function of the *PAX6* mutation carriers and the control members in the families through glucose tolerance test, insulin release test, and glycosylated hemoglobin test.

## 3 RESULTS

### 3.1 Genetic findings

#### 3.1.1 Family 1

**Figure 6:**
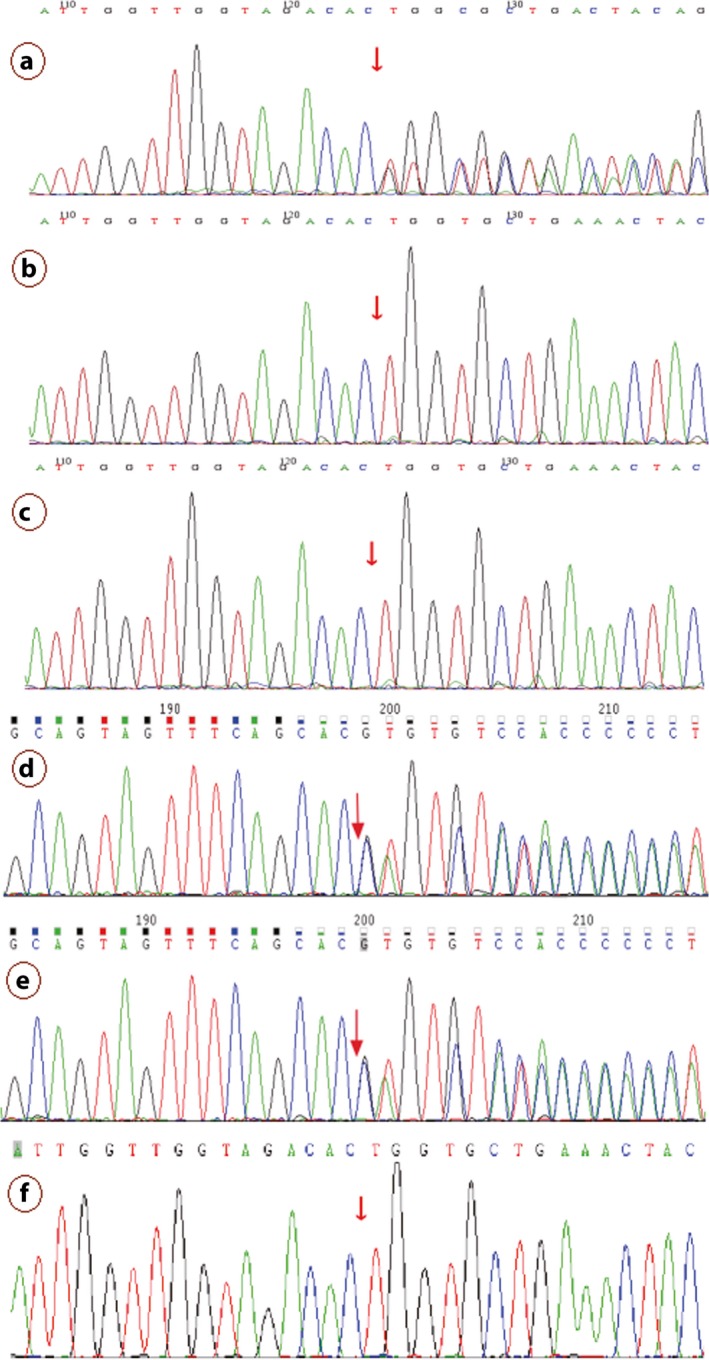
c.879_880del mutation of the *PAX6* validated by Sanger sequencing. Note: Since Sanger validation through forward sequencing or reverse sequencing, the base of the peak map may be the reverse complementation sequence of the base detected. a, b, c, d, e, and f represent the proband (III5), mother(II1), father(II2), the patient(IV1), the patient(III2) and her husband(III1), respectively, in the Family 1

**Figure 7:**
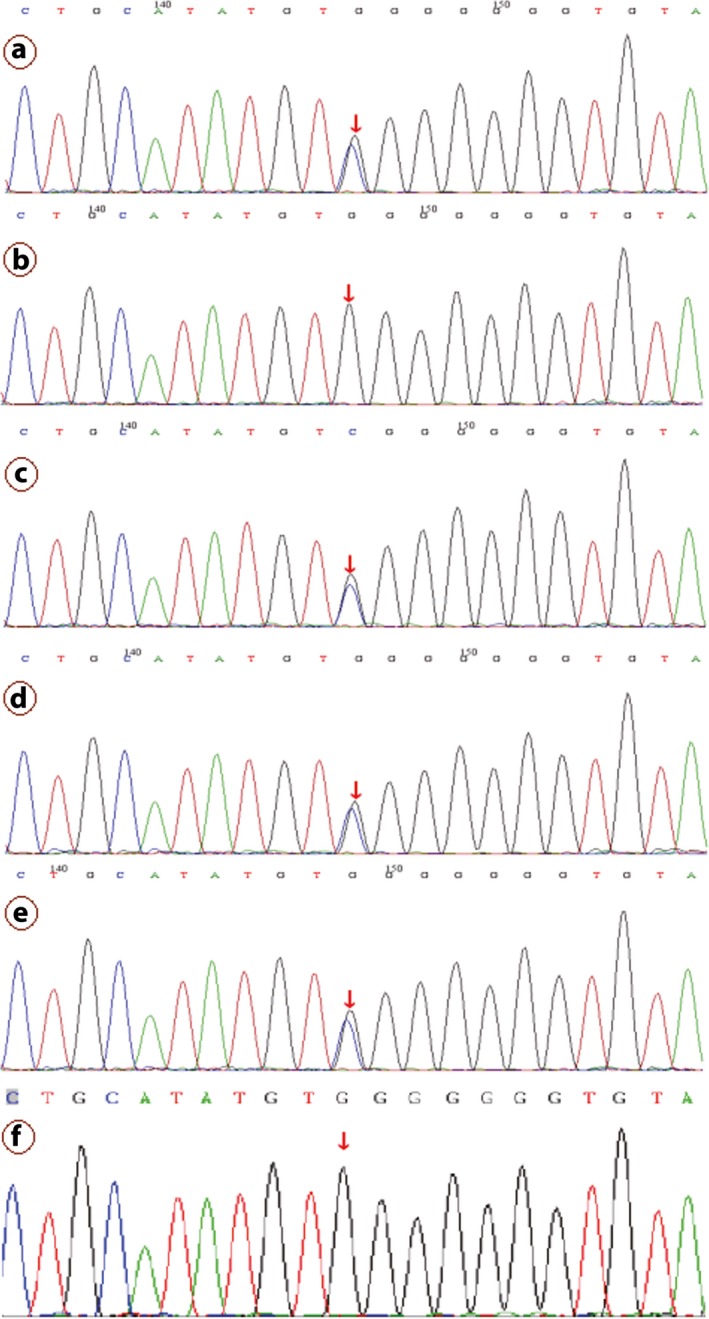
c.1124C>G mutation of the *PAX6* validated by Sanger sequencing. Note: a, b, c, d, e, and f represent the proband (III5), mother(II1), father(II2), the patient(IV1), the patient(III2), and her husband(III1), respectively, in the Family 1

**Figure 8:**
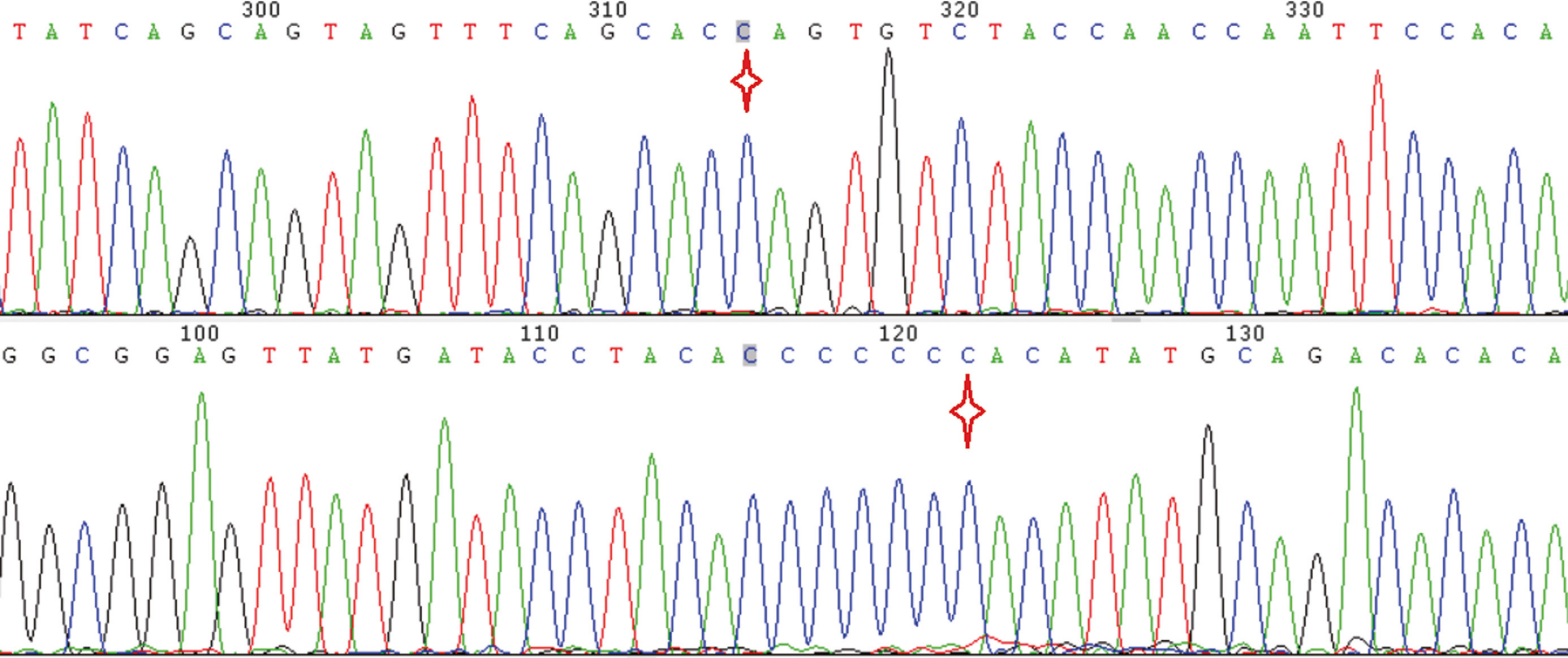
The Sanger sequencing results of the c.879_880 and c.1124 sites of *PAX6* of the sister (III3) with normal phenotypes in the Family 1. Note: Since Sanger validation through forward sequencing or reverse sequencing, the base of the peak map may be the reverse complementation sequence of the base detected

Two related mutations were detected through gene panel sequencing of the proband. We identified a heterozygous frameshift mutation and a heterozygous missense mutation, c.879_880delCA (p.S294Cfs*46) and c.1124C>G (p.P375R) in exon 10 and exon 12 of the *PAX6* gene in proband (III5), respectively, which were confirmed by Sanger sequencing (Figures 6 and  7). No likely pathogenic mutation was detected in the other eye disease genes captured using the NGS panel. We tested and verified the two mutations in nuclear members of Family 1 (III5, II1, II2, IV1, III2, and III1) (Figures 6 and  7). We found that all the three patients (III5, III2, and IV1) have the same two heterozygous mutations that c.879_880delCA (p.S294Cfs*46) and c.1124C>G (p.P375R) of the *PAX6* gene, and the father (II2) just has the c.1124C>G (p.P375R) heterozygous mutation. The other members (III3, III4, III6, IV2, IV3, and IV4) of the Family 1 are without any of the two mutations, and the Sanger sequencing result of the two points of the sister (III3) is shown in Figure 8.

#### 3.1.2 Family 2

**Figure 9:**
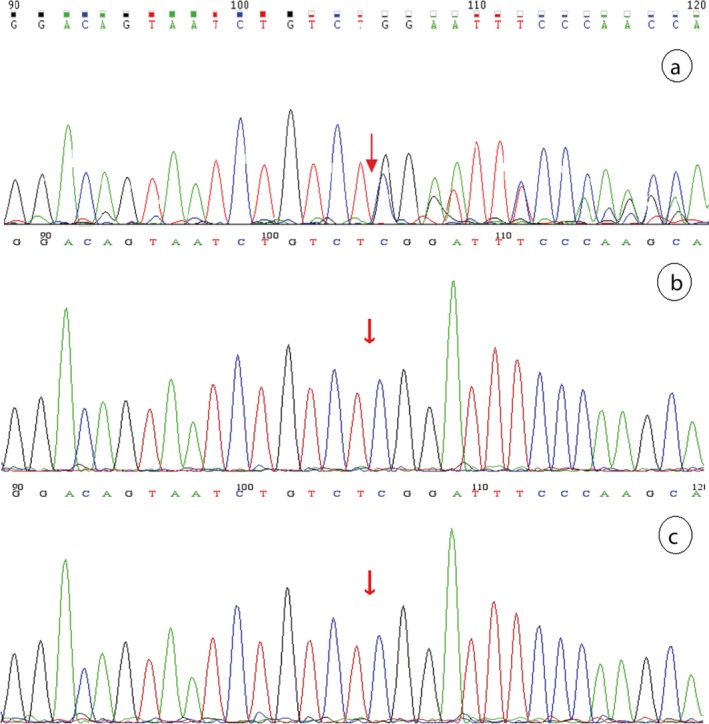
Sanger sequencing result of the *PAX6* c.308delG (p.P103Qfs*21) site in the Family 2. Note: a, b, and c stand for the patient (II1), the patient's mother (I2), and father (I1) in the Family 2, respectively

We identified one heterozygous frameshift mutation, c.308delG (p.P103Qfs*21) of the *PAX6* gene, in the proband in Family 2 through NGS panel sequencing. The patient's parents are both not carried with the mutation tested by the Sanger sequencing experiment (Figure 9).

#### 3.1.3 Family 3

**Figure 10:**
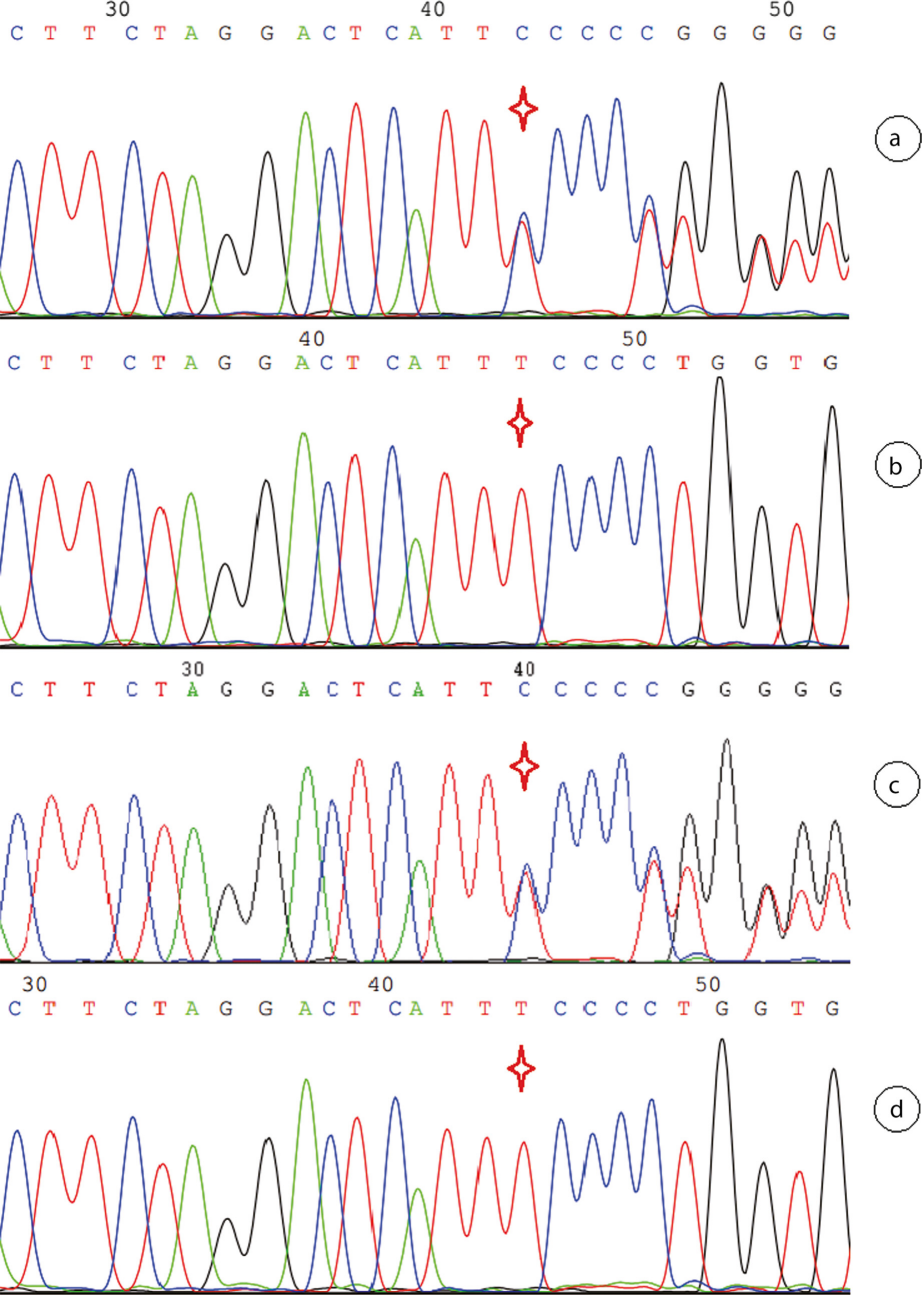
Sanger sequencing result of the *PAX6* c.1192delT (p.S398Pfs*126) site in the Family 3. Note: Red star indicates the mutation site. a, b, c, and d stand for the proband's mother (I2), the proband's father (I1), the proband (II1), and the fetus (II2) in the Family 3, respectively

We found one heterozygous mutation, c.1192delT (p.S398Pfs*126) of the *PAX6* gene, in the proband by NGS sequencing and proved it by Sanger sequencing in all the members of Family 3 (Figure 10), which is a frameshift mutation that not truncated the product but prolonged it. The proband's mother (I2) was the same aniridia patient who also had this heterozygous mutation. We also made a successful prenatal diagnosis of the pregnant woman (II2, Figure 5) by Sanger sequencing of the mutated site, and the fetus proved not to carry with the frameshift mutation and to be a healthy infant after birth.

### 3.2 Pathogenicity analysis

**Figure 11:**
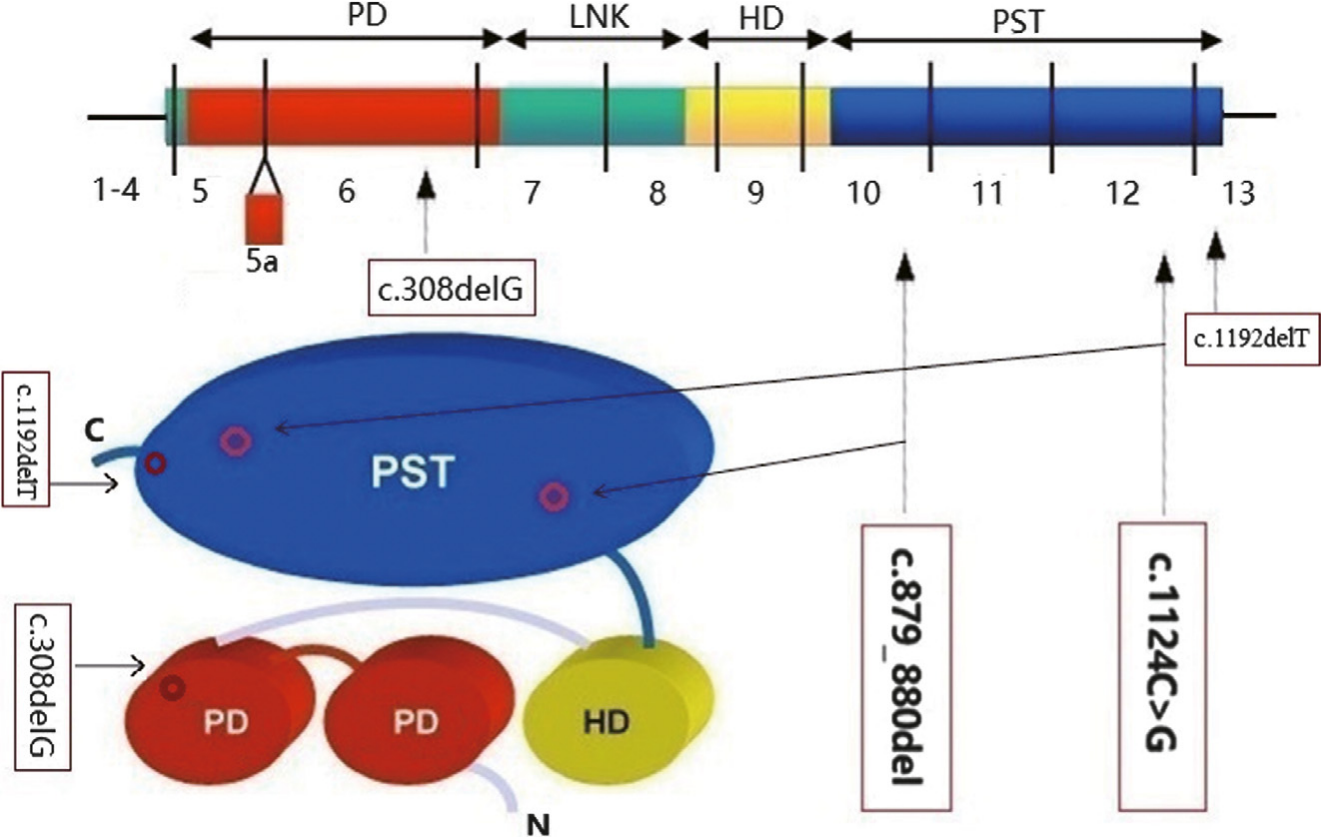
Structural diagrammatic sketch of the PAX6 protein and the four novel mutations position on it. Note: PD is short for paired domain; HD is short for homeodomain; PST is short for proline, serine, and threonine‐rich domain. The picture was made by the Photoshop software

The four mutations are listed in Table 3, which discovered from three congenital aniridia families in this research. These four mutations are all not reported by previous studies or recorded in the public database. Thus, these mutations are de novo discovery in our investigation. And all of the mutations do not appear in the population control of 875 samples collected for this study. So the variants do not belong to the polymorphic loci and their frequencies are extremely low in the population. The position of the mutations in the protein structure is shown in Figure 11. The four mutations are on the paired domain and homeodomain, respectively.

**Table 3:** A survey of the novel mutation sites of *PAX6*

Base position and variation	Protein effect	Results	Rates in family (patients; control)	Control population (WES; panel; WGS) rates	Detection method
c.879_880delCA	p.(S294Cfs*46)	Truncated	3/3; 0/9	0/22; 0/44; 0/809	Sanger Sequencing, NGS
c.1124C>G	p.(P375R)	Missense	3/3; 1/9	0/22; 0/44; 0/809	Sanger Sequencing, NGS
c.308delG	p.(P103Qfs*21)	Truncated	1/1; 0/2	0/22; 0/44; 0/809	Sanger Sequencing, NGS
c.1192delT	p.(S398Pfs*126)	Prolonged	2/2; 0/2	0/22; 0/44; 0/809	Sanger Sequencing, NGS

The c.1192delT variant leads to prolongation of amino sequence of the product, which original length is 422aa.

There are three frameshift mutations found in our study. The *PAX6* c.879_880delCA and c.308delG are frameshift mutations, which leads to truncated polypeptide that terminated after 46 codons and 21 codons of their mutated points, respectively. According to the classification criteria and guidelines for genetic variation of ACMG (the American College of Medical Genetics and Genomics), the frameshift mutation is most likely pathogenic when the disease caused by loss‐of‐function mutation. So, the *PAX6* c.879_880delCA and c.308delG are at PVS1 grade, which is the highest pathogenic grade of classification of pathogenic variation. The *PAX6* c.1192delT is a frameshift mutation that resulted in one prolonged peptide chain. Loss of function of the *PAX6* gene may also be caused by extension of 102 amino acids of the final product.

**Figure 12:**
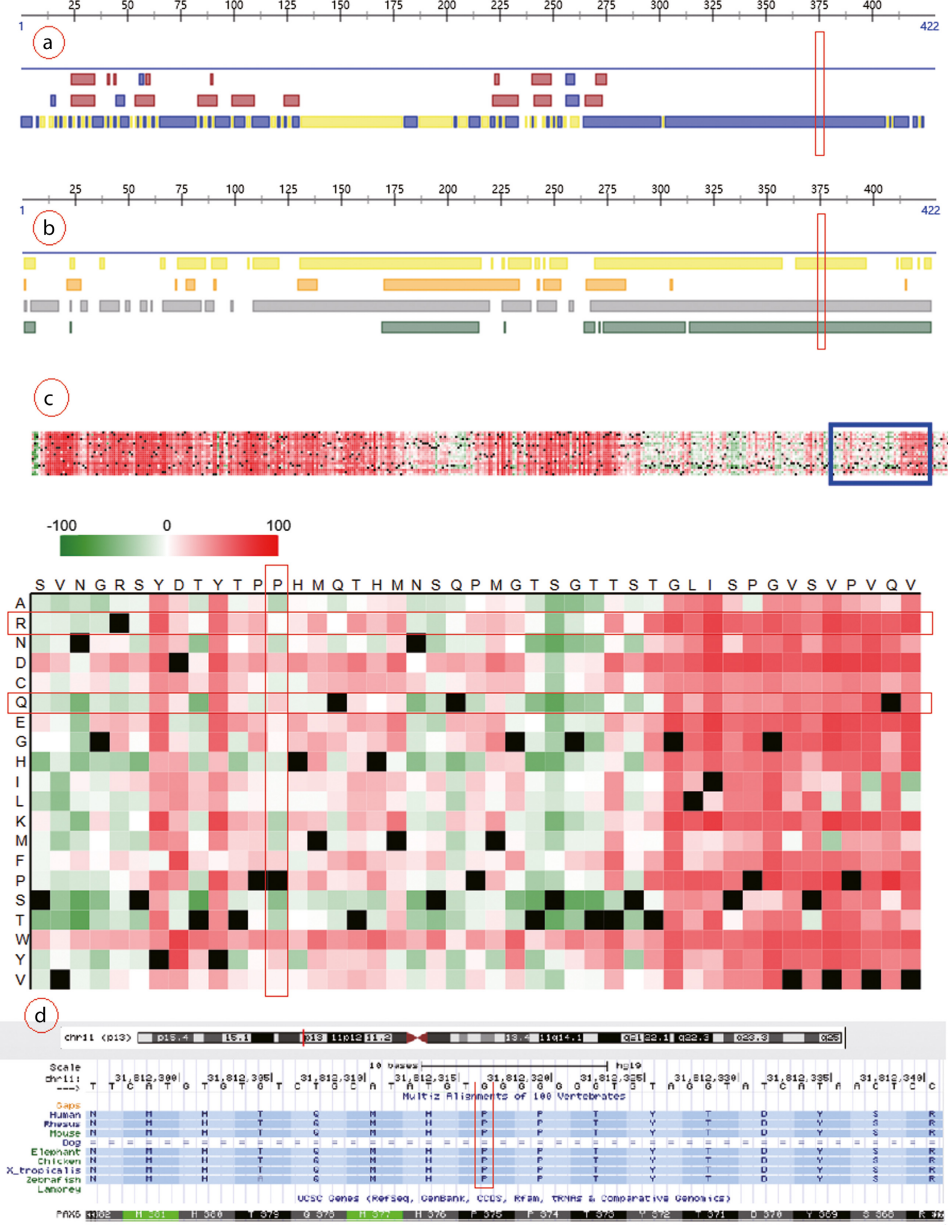
Prediction analysis of the *PAX6* c.1124C>G (p.P375R) missense mutation. Note: Part a presents the result of structure prediction; the mutated site hit third row in which yellow block indicated buried and blue block indicated exposed that calculated by PROF algorithm. Part b presents the result of disorder prediction; the color blocks indicate disorder region and the four rows were calculated by methods of PROFbval, Ucon, NORSnet, and MD, respectively. Part c presents the point effect of c.1124C>G (p.P375R); the horizontal axis and vertical axis stand for normal amino acid sequence and amino acid replacements, the dark red, green, and black indicate damaging, benign, and wild type, respectively. Part d presents the conservation of amino acid sequences corresponding to c.1124C>G mutation of *PAX6* between species

The *PAX6* c.1124C>G is a missense mutation, which leads to proline replaced by arginine at 375th site of the polypeptide chain. Proline is nonpolar heterocyclic amino acid which tends to organize in β‐turn. In contrast, arginine is polar positive charged aliphatic amino acid which has no inclination in secondary structure. This point mutation is predicted to be damaging and disease causing with scores 0.003 and 1 by protein prediction softwares SIFT and MutationTaster, respectively. But it is predicted to be benign with score 0.295 by another tool, PolyPhen_2. The prediction of this missense point by PredictProtein software is shown in Figure 12. We can see that the 375th site of the protein is exposed and in disorder region calculated by methods of PROFbval, Ucon, and MD. The p.P375R substitution score is ‐1, which marked by white color in Part C of Figure 12. And the p.375Q pathogenic reported by previous study is with substitution score 19, which marked by light red color. The higher substitution score is, the more damaging is. The predictive analysis shows that the point mutated effect of p.P375R is milder than p. P375Q. The *PAX6* c.1124C>G site is highly conserved among species, but it is not co‐segregated with the aniridia (Table 3). For these reasons, the *PAX6* c.1124C>G most likely does not lead to congenital aniridia.

### 3.3 Discovery and verification of male gonadal mosaicism

Segregation analysis revealed the mutation, *PAX6* c.879_880delCA, in heterozygous state in the aniridia family members (III2, III5, and IV1), but not in the healthy family members (II1, II2, and III1). But the mutation, *PAX6* c.1124C>G, is not co‐segregation from the aniridia and control of this family due to subject II2 with this mutation but without aniridia phenotype. Another six members of this big family had been conducted Sanger sequencing of the two mutation sites, and none of them carried the mutated sites (Table 1). According to the Sanger sequencing result of Family 1 and the diploidy of human genome, we can deduce that the two mutations of *PAX6* in the patient (IV1) were inherited from his mother (III2) suffered from the same disease. So we can know that these two mutations are linked inherited on one same chromosome 11. In consideration of that the same spontaneous mutation, c.879_880delCA occurred twice and the *PAX6* c.1124C>G mutation of patients (III2 and III5) were reasoned to inherit from their father (II2), so their father is deduced to be existence of mosaic of the gonad probably (Figure 1).

**Figure 13:**
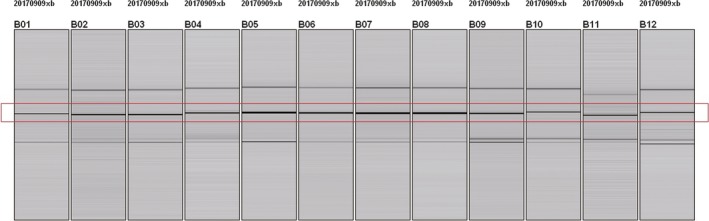
Fast electrophoresis of the amplification product of the *PAX6* target fragments. B01 to B03 are target fragments of exon10 that DNA extracted from blood of proband's father (II2, Family 1); B04 to B06 are target fragments of exon10 that DNA extracted from semen of proband's father (II2, Family 1); B07 to B09 are target fragments of exon10 that DNA extracted from blood of proband (III5, Family 1). Their TMs were set as 56°C,58°C, and 61°C, respectively. B10 to B12 are target fragments of exon12 that DNA extracted from blood of proband's father (II2, Family 1), semen of proband's father (II2, Family 1), and blood of proband (III5, Family 1), respectively. The TM was set as 62°C

**Figure 14:**
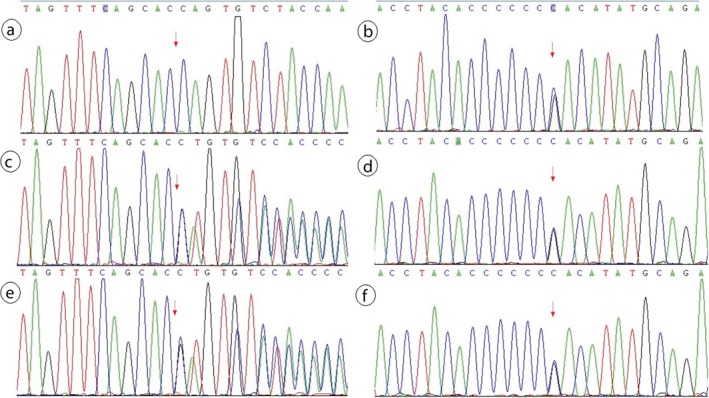
Male gonad chimera(*PAX6*: c.879_880del,c.1124C>G)verified by Sanger sequencing. Note: Parts a and b are the *PAX6* mutation type in blood of proband's father (II2, Family 1); Parts c and d are the *PAX6* mutation type in semen of proband's father (II2, Family 1); Parts e and f are the *PAX6* mutation type in blood of proband (III5, Family 1)

The genetic relationship between parents (II2 and II1) and daughters (III2 and III5) was confirmed by paternity test (Supporting information Figure S1). The electrophoresis results of PCR products of the semen DNA are shown in Figure 13. The Sanger sequencing revealed that the two mutations of *PAX6* both consisted in the sperms of the father and the blood of the proband, but just *PAX6* c.1124C>G is detected in the blood of the father (Figure 14). This experiment result supported the deduction of male mosaic gonad.

### 3.4 Survey of Islet function of the subjects

Glucose tolerance test, insulin release test, and glycosylated hemoglobin test were conducted on members of the three congenital aniridia families, and the results were shown in Table 4. We can discover that most of the patients with isolated aniridia have different degrees of islet damage, except the 2 years old boy. The control members in the families are not observed abnormal indexes.

**Table 4:** Glucose tolerance and insulin release tests of the three families of congenital aniridia

Family	Member	Age (y)	Sex	Aniridia	FPG (mmol/L)	2hPG (mmol/L)	INS (μU/ml)	2hINS (μU/ml)	HbA1c (%)
1	II1	56	M	No	4.52	5.86	9.63	30.21	5.6
	II2	56	F	No	5.61	6.92	8.92	29.72	6.2
	III1	33	M	No	5.17	5.51	6.91	17.59	5.2
	III2^#^	32	F	Yes	5.23	9.73↑	7.05	61.20	6.8↑
	III3	27	F	No	5.45	6.90	5.74	19.32	5.1
	III5^#^	26	F	Yes	8.01↑	10.5↑	11.91↑	20.33	8.9↑
	IV1^#^	13	M	Yes	4.97	7.9↑	6.98	30.67	6.5↑
2	I1	28	M	No	4.93	7.12	6.92	15.65	5.9
	I2	29	F	No	4.82	6.94	7.43	20.36	6.1
	II1^#^	2	M	Yes	4.81	‐	2.96	27.45	‐
3	I1	38	M	No	4.15	6.10	10.10	35.56	6.0
	I2^#^	37	F	Yes	7.32↑	9.12↑	‐	‐	‐
	II1^#^	14	F	Yes	5.95	8.06↑	8.5	59.5	6.36

The members labeled by # are patients with aniridia. fasting plasma glucose (FPG) reference value 3.6‐6.1 mmol/L; 2 hr postprandial plasma glucose (2hPG) reference value 3.9‐7.8 mmol/L; children fasting INS (insulin) reference value 2.5‐7.1 μU/ml; adult fasting INS (insulin) reference value 2.2‐11.6 μU/ml; HbA1c (HbA1c) reference value 4%‐6.5%.

## 4 DISCUSSION

Using targeted sequencing technology, we investigated the mutation profile and clinical manifestation in three Chinese families with congenital aniridia. We detected four novel *PAX6* mutations totally in the aniridia cases, which included one missense and three frameshift mutations. All of these mutations were first described, and which acted in three different damaging ways of amino acid replacement, truncated and prolonged of the protein product of *PAX6*. To date, a total of 319 pathogenic variants in the *PAX6* gene have been recorded as congenital aniridia‐causing mutation in HGMD. The paired box 6 is a homeobox and paired domain‐containing protein that acted as a highly conserved transcriptional regulator which is important for normal ocular development (Tzoulaki, White, & Hanson, 2005). In this study, all of the frameshift mutations are predicted to product nonfunctional or damaged protein.

The *PAX6* gene has 422 amino acids of the product of its most common transcript (NM_000280). The *PAX6* gene belongs to a family of genes that play a critical role in the formation of tissues and organs during embryonic development. It encodes homeobox and paired domain‐containing protein‐bounded DNA and functions as a regulator of transcriptional process, which is key in the development of eye (Ton et al., 1991). During embryonic development, the PAX6 protein is thought to activate genes involved in the formation of the eyes, central nervous system, and the pancreas (Fernald, 2004). Additionally, researchers believe that the PAX6 protein controls many aspects of eye development and regulates the expression of various genes related to ocular structures before and after birth (Davis et al., 2008). The pathogenic mutations of *PAX6* can result in effect of haploinsufficiency as the main mechanism leading to various ocular defects (Vincent, Pujo, Olivier, & Calvas, 2003). The *PAX6* is important for islet cells, and it mainly participates in insulin synthesis and secretion through regulating key proteins in the transcription process (Gosmain et al., 2012).

We detected two novel mutations in the Family 1: One is pathogenic and the other may be polymorphism. It is well accepted that the frameshift mutation c.879_880delCA (p.S294Cfs*46) can cause congenital aniridia, which leaded to a truncated protein. In reviewing previous literatures, we found that there are two frameshift mutations reported pathogenic at the same genome coordinate, which are c.879delC (p.Ser294Valfs*71) and c.879dupC (p.T293fsX47) (Vasilyeva et al., 2017; Villarroel et al., 2008). According to the *ACMG STANDARD AND GUIDELINES 2015* (Richards et al., 2015), the frameshift variants at c.879 of *PAX6* are pathogenic and its pathogenicity evidence grade PVS1 is high. These pathogenic mutations indicate that the neighboring sites of c.879 of *PAX6* are at the hotspot mutation region. The other novel mutation c.1124C>G (p.P375R) is likely to be polymorphic due to its not coseparation from the aniridia and controls. Previous study revealed that the mutation c.1124C>A (p.P375Q) can negatively modulate the homeodomain function in the congenital aniridia patients (Singh, Chao, Mishra, Davies, & Saunders, 2001). From the perspective of inclination of formation of protein secondary structure, the Proline and Glutamine are inclined to constitute β‐turn and α‐helix, and the Arginine has no clear tendency. So the p.P375R mutation of *PAX6* is likely to be benign for congenital aniridia. We found that the c.879_880delCA and c.1124C>G of *PAX6* are double mutants linked hereditary. Retrieving previous researches, we found several cases of double mutation in other genes and diseases (Ainsworth & Coulter‐Mackie, 1992; Hong, Ohashi, Yu, Weiler, & Barranger, 1990; Jensen et al., 1997), and the double mutation is no seen in *PAX6* from previous public publications. Taking advantage of the double mutation of *PAX6* skillfully, the male reproductive gland chimerism was first proved by molecular experiments. We detected two pathogenic novel mutations in the Family 2 and Family 3. According to the *ACMG STANDARD AND GUIDELINES 2015* (Richards et al., 2015), the frameshift variant c.308delG (p.P103Qfs*21) of *PAX6* is pathogenic and its pathogenicity evidence grade PVS1 that is high. It is rare that prolongation of protein causing by frameshift mutation, and it was not reported in *PAX6* by previous studies. We retrieved one case that a deletion mutation of insulin (*INS*) gene predicted to prolong amino acid sequence which rarely occurred in patients with maturity onset diabetes of the young (MODY) (Dusatkova et al., 2015). The mutation c.1192delT (p.S398Pfs*126), in our study, loses the original stop codon resulting in a prolonged protein molecule in this study, which contains an additional 102 amino acids. The redundant repeat sequence after p.S398 may affect the function of the normal protein product. It is acceptive that the frameshift mutation c.1192delT (p.S398Pfs*126) belongs to damaging variant, which produces a prolonged protein with loss of function.

Our islet function test results showed that the aniridia patients frequently also have islet damage, except for the young children in our tests. This may be a normal phenomenon owing to early stage of the disease. The insulin‐related tests on the three families increased their clinical data for comprehensive understanding of the mutations, and it is meaningful for health supervision.

In summary, we investigated the clinical and genetic characteristics of three families of congenital aniridia in China. We reported three novel pathogenic mutations for aniridia, which lead to two different types of effect of truncated or prolonged proteins. We also reported one novel missense mutation of undetermined significance. To our knowledge, this is the first discovery and confirmation of male reproductive gland chimerism in aniridia by logical reasoning and Sanger sequencing. Our results extend the mutation spectrum of *PAX6* and related clinical phenotypes, which has guiding significance for the diagnosis and prenatal diagnosis of congenital aniridia.

## CONFLICT OF INTEREST

The authors report no conflict of interest.

## References

[mgg3481-bib-0001] Ainsworth, P. J. , & Coulter‐Mackie, M. B. (1992). A double mutation in exon 6 of the beta‐hexosaminidase alpha subunit in a patient with the B1 variant of Tay‐Sachs disease. American Journal of Human Genetics, 51(4), 802–809. 1415222 PMC1682773

[mgg3481-bib-0002] Davis, L. K. , Meyer, K. J. , Rudd, D. S. , Librant, A. L. , Epping, E. A. , Sheffield, V. C. , & Wassink, T. H. (2008). Pax6 3’ deletion results in aniridia, autism and mental retardation. Human Genetics, 123(4), 371–378. 10.1007/s00439-008-0484-x 18322702 PMC2719768

[mgg3481-bib-0003] Dusatkova, L. , Dusatkova, P. , Vosahlo, J. , Vesela, K. , Cinek, O. , Lebl, J. , & Pruhova, S. (2015). Frameshift mutations in the insulin gene leading to prolonged molecule of insulin in two families with Maturity‐Onset Diabetes of the Young. European Journal of Medical Genetics, 58(4), 230–234. 10.1016/j.ejmg.2015.02.004 25721872

[mgg3481-bib-0004] Fernald, R. D. (2004). Eyes: Variety, development and evolution. Brain, Behavior and Evolution, 64(3), 141–147. 10.1159/000079743 15353906

[mgg3481-bib-0005] Glaser, T. , Jepeal, L. , Edwards, J. G. , Young, S. R. , Favor, J. , & Maas, R. L. (1994). PAX6 gene dosage effect in a family with congenital cataracts, aniridia, anophthalmia and central nervous system defects. Nature Genetics, 7(4), 463–471. 10.1038/ng0894-463 7951315

[mgg3481-bib-0006] Gosmain, Y. , Katz, L. S. , Masson, M. H. , Cheyssac, C. , Poisson, C. , & Philippe, J. (2012). Pax6 is crucial for beta‐cell function, insulin biosynthesis, and glucose‐induced insulin secretion. Molecular Endocrinology, 26(4), 696–709. 10.1210/me.2011-1256 22403172 PMC5417143

[mgg3481-bib-0007] Hong, C. M. , Ohashi, T. , Yu, X. J. , Weiler, S. , & Barranger, J. A. (1990). Sequence of two alleles responsible for Gaucher disease. DNA and Cell Biology, 9(4), 233–241. 10.1089/dna.1990.9.233 1972019

[mgg3481-bib-0008] Jensen, H. K. , Jensen, T. G. , Faergeman, O. , Jensen, L. G. , Andresen, B. S. , Corydon, M. J. , … Gregersen, N. (1997). Two mutations in the same low‐density lipoprotein receptor allele act in synergy to reduce receptor function in heterozygous familial hypercholesterolemia. Human Mutation, 9(5), 437–444. 10.1002/(ISSN)1098-1004 9143924

[mgg3481-bib-0009] Lim, H. T. , Kim, D. H. , & Kim, H. (2017). PAX6 aniridia syndrome: Clinics, genetics, and therapeutics. Current Opinion in Ophthalmology, 28(5), 436–447. 10.1097/ICU.0000000000000405 28598868

[mgg3481-bib-0010] Richards, S. , Aziz, N. , Bale, S. , Bick, D. , Das, S. , Gastier‐Foster, J. , … Rehm, H. L. (2015). Standards and guidelines for the interpretation of sequence variants: A joint consensus recommendation of the American College of Medical Genetics and Genomics and the Association for Molecular Pathology. Genetics in Medicine, 17(5), 405–424. 10.1038/gim.2015.30 25741868 PMC4544753

[mgg3481-bib-0011] Schlessinger, A. , Yachdav, G. , & Rost, B. (2006). PROFbval: Predict flexible and rigid residues in proteins. Bioinformatics, 22(7), 891–893. 10.1093/bioinformatics/btl032 16455751

[mgg3481-bib-0012] Singh, S. , Chao, L. Y. , Mishra, R. , Davies, J. , & Saunders, G. F. (2001). Missense mutation at the C‐terminus of PAX6 negatively modulates homeodomain function. Human Molecular Genetics, 10(9), 911–918. 10.1093/hmg/10.9.911 11309364

[mgg3481-bib-0013] Ton, C. C. , Hirvonen, H. , Miwa, H. , Weil, M. M. , Monaghan, P. , Jordan, T. , … Saunders, G. F. (1991). Positional cloning and characterization of a paired box‐ and homeobox‐containing gene from the aniridia region. Cell, 67(6), 1059–1074. 10.1016/0092-8674(91)90284-6 1684738

[mgg3481-bib-0014] Tzoulaki, I. , White, I. M. , & Hanson, I. M. (2005). PAX6 mutations: Genotype‐phenotype correlations. BMC Genetics, 6, 27 10.1186/1471-2156-6-27 15918896 PMC1156885

[mgg3481-bib-0015] Vasilyeva, T. A. , Voskresenskaya, A. A. , Kasmann‐Kellner, B. , Khlebnikova, O. V. , Pozdeyeva, N. A. , Bayazutdinova, G. M. , … Zinchenko, R. A. (2017). Molecular analysis of patients with aniridia in Russian Federation broadens the spectrum of PAX6 mutations. Clinical Genetics, 92(6), 639–644. 10.1111/cge.13019 28321846

[mgg3481-bib-0016] Villarroel, C. E. , Villanueva‐Mendoza, C. , Orozco, L. , Alcantara‐Ortigoza, M. A. , Jimenez, D. F. , Ordaz, J. C. , & Gonzalez‐del Angel, A. (2008). Molecular analysis of the PAX6 gene in Mexican patients with congenital aniridia: Report of four novel mutations. Molecular Vision, 14, 1650–1658. 18776953 PMC2530489

[mgg3481-bib-0017] Vincent, M. C. , Pujo, A. L. , Olivier, D. , & Calvas, P. (2003). Screening for PAX6 gene mutations is consistent with haploinsufficiency as the main mechanism leading to various ocular defects. European Journal of Human Genetics, 11(2), 163–169. 10.1038/sj.ejhg.5200940 12634864

