# Supplementary material for: Extension of the mutation spectrum of PAX6 from three Chinese congenital aniridia families and identification of male gonadal mosaicism
Source: Mol Genet Genomic Med. 2018 Oct 17;6(6):1053–67. doi: 10.1002/mgg3.481 (PMC6305634; doi:10.1002/mgg3.481)
Supplement: Supplementary file 1 [file MGG3-6-1053-s001.docx]

Supplementary Material

Table S1. Gene list of the NGS penal

| ABCA4 | ABCB6 | ABCC6 | ABHD12 | ACBD5 | ACO2 |
| --- | --- | --- | --- | --- | --- |
| ADAM9 | ADAMTS10 | ADAMTS17 | ADAMTS18 | ADAMTSL4 | ADAR |
| ADIPOR1 | AGBL1 | AGBL5 | AGK | AGPS | AHI1 |
| ALDH1A3 | ALMS1 | ANO5 | ANTXR1 | AP3B1 | APOE |
| ARL2BP | ARL3 | ARL6 | ARMS2 | ASB10 | ASRGL1 |
| ATOH7 | ATP13A2 | ATP1A3 | ATP2C1 | ATXN10 | ATXN7 |
| BBIP1 | BBS1 | BBS10 | BBS12 | BBS2 | BBS4 |
| BBS9 | BCOR | BEST1 | BFSP1 | BFSP2 | BLOC1S3 |
| C10orf11 | C12orf57 | C12orf65 | C1QTNF5 | C2 | C21orf2 |
| C5AR2 | C5orf42 | C8orf37 | C9 | CA4 | CABP4 |
| CACNA2D4 | CAPN3 | CAPN5 | CASK | CAV1 | CAV3 |
| CDH23 | CDH3 | CDHR1 | CEP164 | CEP250 | CEP290 |
| CERKL | CFB | CFH | CFHR1 | CFHR3 | CFI |
| CHM | CHMP4B | CHN1 | CHST6 | CIB2 | CISD2 |
| CLN5 | CLN6 | CLN8 | CLRN1 | CLUAP1 | CNBP |
| CNGB1 | CNGB3 | CNNM4 | CNOT9 | COL11A1 | COL11A2 |
| COL4A1 | COL6A1 | COL6A2 | COL6A3 | COL7A1 | COL8A2 |
| CRB1 | CRX | CRYAA | CRYAB | CRYBA1 | CRYBA2 |
| CRYBB2 | CRYBB3 | CRYGA | CRYGB | CRYGC | CRYGD |
| CST3 | CTC1 | CTDP1 | CTNNA1 | CTNND2 | CTSD |
| CYP1B1 | CYP27A1 | CYP4V2 | CYP51A1 | DCN | DHCR7 |
| DMD | DMPK | DNA2 | DNAJC5 | DNM1L | DOCK9 |
| DTHD1 | DTNBP1 | DUX4 | DYSF | EFEMP1 | ELOVL4 |
| EMD | EPG5 | EPHA1 | EPHA2 | ERBB3 | ERCC2 |
| ERCC5 | ERCC6 | ERCC8 | ESR1 | EXOSC2 | EYA1 |
| FAM126A | FAM161A | FBLN5 | FBN1 | FBN2 | FGFR3 |
| FKTN | FLVCR1 | FOXC1 | FOXD3 | FOXE3 | FOXL2 |
| FRMD7 | FSCN2 | FTL | FTO | FXN | FYCO1 |
| GALT | GCNT2 | GDF3 | GDF6 | GFER | GJA1 |
| GJB1 | GJB2 | GLI2 | GMPPB | GNAT1 | GNAT2 |
| GNPTG | GP1BA | GPR143 | GPR179 | GRK1 | GRM6 |
| GSTM1 | GSTT1 | GUCA1A | GUCA1B | GUCY2D | GUSB |
| HDAC6 | HESX1 | HEXA | HEXB | HFE | HGF |
| HMCN1 | HMGB3 | HMX1 | HPS1 | HPS3 | HPS4 |
| HSF4 | HTRA1 | IARS2 | IDH3B | IDO1 | IDUA |
| IFT172 | IFT27 | IGBP1 | IMPDH1 | IMPG1 | IMPG2 |
| IQCB1 | IRX5 | ITGA2B | ITGA7 | ITGB3 | ITM2B |
| KCNV2 | KCTD7 | KERA | KIAA1549 | KIF11 | KIF21A |
| KLC2 | KLHL7 | KMT2D | KRT12 | KRT3 | LAMA1 |
| LCA5 | LCAT | LCT | LGR4 | LIM2 | LMNA |
| LRAT | LRIT3 | LRP5 | LRPAP1 | LTBP2 | LYST |
| MAF | MAK | MAN2B1 | MANBA | MAPKAPK3 | MAPT |
| MERTK | MFN2 | MFRP | MFSD8 | MIP | MITF |
| MLPH | MPDZ | MPV17 | MRE11 | MTHFR | MTM1 |
| MT-ND4 | MT-ND4L | MT-ND5 | MT-ND6 | MT-TI | MT-TL1 |
| MTTP | MVK | MYH2 | MYH7 | MYH9 | MYO5A |
| MYOT | NAA10 | NAT2 | NBAS | NDP | NEB |
| NEK2 | NEUROD1 | NF1 | NF2 | NHS | NMNAT1 |
| NPHP1 | NPHP3 | NPHP4 | NR2E1 | NR2E3 | NR2F1 |
| NXNL1 | NYX | OAT | OCA2 | OCRL | OFD1 |
| OPA3 | OPA6 | OPN1LW | OPN1MW | OPN1MW2 | OPN1SW |
| OR2W3 | OTX2 | P3H2 | P3H3 | P3H4 | PABPN1 |
| PAX3 | PAX6 | PCDH15 | PCYT1A | PDCD2 | PDE6A |
| PDE6D | PDE6G | PDE6H | PDZD7 | PEX1 | PEX10 |
| PEX13 | PEX14 | PEX16 | PEX19 | PEX2 | PEX26 |
| PEX5L | PEX6 | PEX7 | PGK1 | PGR | PHOX2A |
| PIKFYVE | PITPNM3 | PITX2 | PITX3 | PLA2G5 | PLEKHA1 |
| PNPLA6 | POC1B | POLG | POLG2 | POMGNT1 | POMT1 |
| PPT1 | PRCD | PRDM13 | PRIMPOL | PRKCG | PROM1 |
| PRPF4 | PRPF6 | PRPF8 | PRPH2 | PRPS1 | PRSS56 |
| RAB27A | RAB28 | RAB3GAP1 | RAB3GAP2 | RAB7A | RABGGTA |
| RAX | RAX2 | RB1 | RBP3 | RBP4 | RCBTB1 |
| RDH12 | RDH5 | RECQL4 | RGR | RGS9 | RGS9BP |
| RLBP1 | RNASEH1 | RNLS | ROBO3 | ROM1 | RP1 |
| RP9 | RPE65 | RPGR | RPGRIP1 | RPGRIP1L | RRM2B |
| SAG | SALL1 | SALL2 | SBF2 | SC5D | SCO2 |
| SEC23A | SELENON | SEMA4A | SGCA | SGCB | SGCD |
| SHOX | SIL1 | SIX5 | SIX6 | SLC16A12 | SLC16A2 |
| SLC25A15 | SLC25A4 | SLC26A4 | SLC2A1 | SLC33A1 | SLC38A8 |
| SLC4A11 | SLC6A5 | SLC7A14 | SLC9A6 | SLITRK6 | SMOC1 |
| SNRNP200 | SOD1 | SORD | SOX10 | SOX2 | SPATA7 |
| SRD5A3 | SREBF2 | STRA6 | SYNE1 | SYNE2 | TACSTD2 |
| TBC1D24 | TBK1 | TBX1 | TCAP | TCF4 | TCOF1 |
| TCTN3 | TDRD7 | TEAD1 | TENM3 | TFAP2A | TGFB3 |
| TGFBR2 | TIMM8A | TIMP3 | TINF2 | TK2 | TLR3 |
| TMEM114 | TMEM126A | TMEM138 | TMEM216 | TMEM231 | TMEM237 |
| TMEM98 | TMX3 | TNNT1 | TOPORS | TPM2 | TPM3 |
| TRNT1 | TRPM1 | TSPAN12 | TTC21B | TTC8 | TTLL5 |
| TTR | TUB | TUBA8 | TUBB3 | TUBGCP4 | TUBGCP6 |
| TYRP1 | UBIAD1 | UCHL1 | UNC119 | UNC45B | USH1C |
| VAX1 | VCAN | VHL | VIM | VLDLR | VPS13B |
| WDPCP | WDR19 | WDR36 | WDR73 | WFS1 | WHRN |
| ZEB1 | ZNF408 | ZNF423 | ZNF469 | ZNF513 | ZNF644 |
| ACTA1 | ADGRA3 | AIPL1 | APTX | ATF6 | B3GLCT |
| ACTB | ADGRV1 | ALDH18A1 | ARL13B | ATM | B9D1 |
| BBS5 | BLOC1S6 | C2orf71 | CACNA1A | CC2D2A | CEP41 |
| BBS7 | BMP4 | C3 | CACNA1F | CCDC28B | CEP78 |
| CFL2 | CLDN19 | CNGA1 | COL18A1 | COL9A1 | CRYBA4 |
| CHD7 | CLN3 | CNGA3 | COL2A1 | COL9A2 | CRYBB1 |
| CRYGS | CTSF | DHDDS | DRAM2 | ELP4 | ERCC3 |
| CSPP1 | CX3CR1 | DHX38 | DRD5 | EMC1 | ERCC4 |
| EYS | FHL1 | FRAS1 | FZD4 | GJA3 | GNB3 |
| EZR | FKRP | FREM2 | GALK1 | GJA8 | GNPAT |
| GRN | HARS | HGSNAT | HPS5 | IFNGR1 | INPP5E |
| GSN | HCCS | HK1 | HPS6 | IFT140 | INVS |
| JAG1 | KIF7 | LAMA2 | LMX1B | LZTFL1 | MC1R |
| JAM3 | KIZ | LARGE1 | LOXHD1 | MAB21L2 | MCOLN1 |
| MKKS | MT-ND1 | MT-TL2 | MYO7A | NECTIN1 | NOD2 |
| MKS1 | MT-ND3 | MT-TN | MYOC | NECTIN3 | NOG |
| NRL | OLFM2 | OPTC | PANK2 | PDE6B | PEX11B |
| NTF4 | OPA1 | OPTN | PAX2 | PDE6C | PEX12 |
| PEX3 | PHYH | PLG | POMT2 | PRPF3 | PXDN |
| PEX5 | PIGL | PLK4 | POU3F4 | PRPF31 | RAB18 |
| RABGGTB | RD3 | RHO | RP1L1 | RS1 | SDCCAG8 |
| RARB | RDH11 | RIMS1 | RP2 | RYR1 | SDHA |
| SGCG | SLC24A1 | SLC39A5 | SMS | SPG7 | TAT |
| SHH | SLC24A5 | SLC45A2 | SNAI2 | SPP2 | TBC1D20 |
| TCTN1 | TGFBI | TLR4 | TMEM67 | TPP1 | TTN |
| TCTN2 | TGFBR1 | TLR6 | TMEM70 | TRIM32 | TTPA |
| TULP1 | USH1G | VSX1 | WRN | TYR | USH2A |
| VSX2 | YAP1 |  |  |  |  |


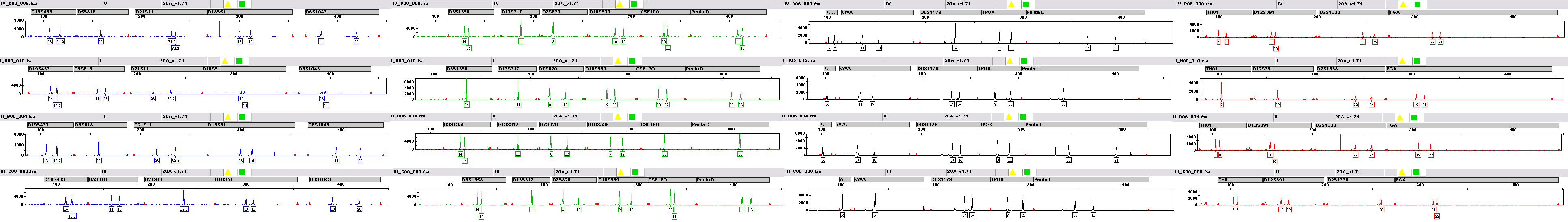


Figure S1 Paternity verification of the Family 1. IV_D06_008 stands for father(II2), I_H05_015 stands for mother(II1), II_B06_004 stands for the suffering sister of the proband(III2), III_C06_006 stands for the proband(III5).
